# Effects of movement velocity in squat training with and without blood flow restriction

**DOI:** 10.5114/biolsport.2026.160853

**Published:** 2026-04-13

**Authors:** Pedro Jesús Cornejo-Daza, Juan Sánchez-Valdepeñas, Luis Rodiles-Guerrero, Paula Costales-Muñoz, Miguel Sánchez-Moreno, Irineu Loturco, Juan A León-Prados, Fernando Pareja-Blanco

**Affiliations:** 1 Science-Based Training Research Group, Physical Performance and Sports Research Center (CIRFD), Universidad Pablo de Olavide, Seville, Spain; 2 Faculty of Human Sciences and Education, Department of Musical, Plastic and Corporal Expression, University of Zaragoza, Huesca, Spain; 3 ENFYRED Research Group, Faculty of Health and Sports Sciences, Department of Musical, Plastic and Corporal Expression, University of Zaragoza, Huesca, Spain; 4 Physical Education and Sports Department, Cardenal Spinola CEU Andalucia University, Bormujos, Seville, Spain; 5 Faculty of Education, Psychology and Sport Sciences, University of Huelva, Spain; 6 Faculty of Sport Sciences, Department of Sports and Computers Sciences. Universidad Pablo de Olavide, Seville, Spain; 7 Department of Physical Education and Sports. University of Seville, Seville, Spain; 8 Nucleus of High Performance in Sport (NAR), São Paulo, Brazil; 9 Department of Human Movement Sciences, Federal University of São Paulo, São Paulo, Brazil; 10 University of South Wales, Pontypridd, Wales, United Kingdom

**Keywords:** Velocity-based training, Intentionality, Maximal velocity, Arterial occlusion pressure, Neural adaptations, Structural adaptations

## Abstract

This study compared the effects of four different resistance training (RT) programs that differed in the movement velocity [maximum (MaxV) vs. 50% of maximum voluntary velocity (HalfV)] and the blood flow condition [free (FF) vs. restricted (BFR)] on strength, neuromuscular, and hypertrophy adaptations. Fortysix resistance-trained males were randomly assigned to one of the four abovementioned protocols. Subjects followed an 8-week RT program, twice per week, with similar intensity (55%–65% 1RM), sets (3), and repetitions per set (10–6) in the full-squat (SQ) exercise. Before and after the RT program, they were evaluated for: 1) muscle size of the vastus lateralis; 2) vertical jump; 3) maximal isometric contraction; 4) progressive loading test; and 5) fatigue test, in SQ. Electromyographic (EMG) activity was assessed during all SQ tests. All protocols exhibited significant gains in strength-derived parameters without significant differences between groups. A BFR × VEL × time interaction (p = 0.05) was observed for countermovement jump, with improvements in the HalfV-BFR and MaxV-FF protocols. A VEL × time interaction (p = 0.03) was found for the EMG amplitude under light loads, since HalfV-BFR increased its values. MaxV-BFR and HalfV-FF induced greater hypertrophy in the distal region of the vastus lateralis (BFR × VEL × time interaction, p = 0.04), with MaxV-BFR producing the greatest gains in all sections. Both MaxV-FF and HalfV-BFR increased CMJ height. HalfV-BFR also increased neuromuscular activity against light loads. MaxV-BFR and HalfV-FF evoked higher hypertrophy in muscle size, with the greatest gains in muscle size with MaxV-BFR.

## INTRODUCTION

Blood flow restriction (BFR) involves applying external pressure to a limb (proximal to the muscles being exercised) with an inflatable cuff to reduce the blood flow to the exercising muscles [1]. Resistance training (RT) with low load (20–40% of the one-repetition maximum [1RM]) [2, 3] performed to volitional fatigue [4, 5] (or close to it) with BFR has been shown to promote increases in muscle strength and hypertrophy [4, 6]. These increases in muscle strength and hypertrophy with low-load BFR are greater than those with lowload free-flow (FF) resistance exercise matched for the same work [7, 8]. Conversely, high-load FF exercise has shown greater muscle strength gains and similar muscle mass increases than lowload BFR-RE and different neural stimuli [9, 10].

On the other hand, movement velocity is one of the most important variables that can be manipulated to regulate and control the mechanical stimulus during RT sessions [11, 12]. Movement velocity depends on the magnitude of the load to be lifted and on the voluntary intention of the athlete to move that load [11, 13]. This variable may influence the adaptations and acute effects resulting from RT, as movement velocity has been shown to be more important than time under tension (TUT) in inducing neuromuscular adaptations aimed at improving athletic performance [14, 15]. Pareja-Blanco et al., [15] analysed the effects of two full-squat (SQ) training programs, which were equivalent in all training variables except movement velocity. The results indicated that performing repetitions at maximal concentric velocity, compared to intentionally slower at halfvelocity, resulted in a likely more beneficial effect on SQ performance and countermovement jump (CMJ) height [15].

Despite the benefits of movement velocity and BFR-RT having been widely documented independently, their combined effects have not yet been thoroughly investigated. Most studies examining BFRRT have employed protocols in which each repetition is performed in a controlled manner [3–5, 7, 8], with only a few investigating repetitions executed at maximal concentric velocity under BFR conditions [16, 17, 18]. Nevertheless, it remains unknown whether manipulating movement velocity while maintaining the same relative load during BFR-RT could influence long-term adaptations, or whether this combination of BFR and velocity manipulation would elicit adaptations similar to those observed in FF-RT. Consequently, the aim of this study was to analyze the effects of four different RT programs in the SQ exercise that differed in movement velocity (maximum voluntary velocity vs. 50% of maximum voluntary velocity) and blood flow condition (FF vs. BFR) on jump performance, muscle strength, neuromuscular adaptations, and muscle hypertrophy.

## MATERIALS AND METHODS

### Experimental Approach to the Problem

A longitudinal study design was implemented to assess the effects of four RT programs, which differed in movement velocity (MaxV vs. HalfV) and blood flow conditions (FF vs. BFR). Subjects underwent two sessions per week, spaced 48 to 72 hours apart, over an 8-week progressive RT program focused exclusively on the SQ exercise. The four protocols shared the same intensity (55–65% 1RM), volume (3 sets of 10, 8, and 6 reps), and rest intervals between sets (2 minutes). The tests were conducted in a research laboratory, under the direct supervision of the research team, and with consistent environmental conditions (20°C and 60% humidity). Subjects were instructed to refrain from engaging in any other strenuous physical activity before testing. All subjects completed a battery of assessments over two testing sessions: 72 hours before (pre-training) and 72 hours after (post-training) the 8-week program. The first testing session involved ultrasound evaluations of the vastus lateralis (VLA) muscle at different femur lengths (40%, 57.5%, and 75%). In the second testing session, several fitness assessments were performed, including: 1) CMJ); 2) Maximal voluntary isometric contraction (MVIC) at 90º in SQ; 3) Progressive loading test in SQ, and 4) Fatigue test in SQ.

### Subjects

The sample size was calculated using G-Power (Version 3.1.9.2, Heinrich-Heine-Universität Düsseldorf, Düsseldorf, Germany) introducing the following parameters: effect size (ES = 0.7 based on ES observed for 1RM in previous literature using a similar approach [19]; and α error probability (0.05) and power (0.95), four groups and two measurements, which resulted in a sample size of 10 subjects per group. A total of fifty-two resistance-trained men participated in the study. All subjects had prior experience with RT (1.5 to 4 years), specifically in the SQ exercise. The subjects were randomly divided into four groups, which varied based on movement velocity (MaxV: maximum voluntary velocity vs. HalfV: 50% of maximum voluntary velocity) and blood flow condition (FF: free flow condition vs. BFR: 50% of arterial occlusion pressure). Six participants discontinued their participation for personal reasons unrelated to the study protocols (e.g., lack of time or work commitments), leaving the final group numbers as follows: MaxV-FF (n = 13), HalfV-FF (n = 12), MaxVBFR (n = 11), HalfV-BFR (n = 10). Thus, the final sample consisted of forty-six subjects (aged 18–38 years, age = 23.2 ± 4.9 years, height = 1.76 ± 0.07 m, body mass = 76.7 ± 13.6 kg; 1RM relative to body mass = 1.40 ± 0.23). The performance level of the subjects was classified under tiers 1 and 2, following the framework from McKay et al. [20]. All participants were informed of the study’s purpose and testing procedures and signed a consent form before participation. The study was approved by the Research Ethics Committee of “Hospitales Universitarios Virgen Macarena-Virgen del Rocío” (Reference: 1547-N-19), in accordance with the Declaration of Helsinki. No participant reported any physical restrictions, health conditions, or injuries that might affect testing, nor were they using drugs, medications, or supplements prior to the testing.

### Testing procedures

#### Ultrasonography

The anatomical cross-sectional area (ACSA) of the VLA muscle in the right leg was measured using B-mode ultrasonography (MyLab 25, Esaote Biomedica, Italy) with a 50-mm, 5–12 MHz linear probe. The VLA muscle is commonly selected in research because of its size and the ease of capturing clear ultrasound images [21]. Participants lay in a supine position with knees flexed at 150° (where 180º represents full knee extension), supported over a foam roller. After 15 minutes in this position, ACSA was measured at 40% (distal; ACSA_40_), 57.5% (medial; ACSA_57.5_), and 75% (proximal; ACSA_75_) of the distance between the femur’s lateral condyle and the top of the greater trochanter (with the distal point considered as 0% [22]. The extended field of view mode was used, and adhesive markers were placed to ensure consistent probe movement. The panoramic image was captured from medial to lateral, moving carefully and at a constant velocity along the defined path, ensuring that the probe remained perpendicular to the surface of the skin and applying minimum pressure to avoid excessive tissue compression. Three images were taken at each site and analyzed using ImageJ software (ImageJ 1.51j8 software, NIH) to calculate ACSA by tracing the muscle’s boundaries (aponeuroses). Two US images were assessed for each variable, and if a coefficient of variation (CV) above 5% was detected, an additional image was examined. Measurements were averaged, and consistency was maintained by marking probe positions on transparent acetate and using identifiable infiltrations of fatty and connective tissues as landmarks. Muscle volume (cm^3^) was estimated using a method based on data from five ACSA slices [23].

#### Vertical jump test

Subjects performed five maximal CMJs, with 20-s rests between each jump. Before the CMJ test, subjects completed a warm-up that included 5 minutes of jogging at a self-selected easy pace, 2 sets of 10 SQs without external load, 5 submaximal CMJs, and 3 maximal CMJs. Subjects were instructed to land upright and bend their knees upon landing. CMJ height was calculated based on flight time, measured using an infrared timing system (OptojumpNext, Microgate, Bolzano, Italy). The highest and lowest jump heights were discarded, and the average of the remaining jumps was used for analysis.

#### Maximal Voluntary Isometric Contraction test

Subjects performed two 5-second maximal isometric contractions in the SQ position with knees flexed at 90° (180° representing full extension), separated by 1 minute of rest. The test was done using a Smith machine with height-adjustable movable supports and an 80 x 80-cm dynamometric platform (FP-500, Ergotech, Murcia, Spain). Subjects were instructed to push against the force platform as fast and hard as possible upon receiving the cue “Ready, set, go!”. External forces were recorded at a sampling rate of 1,000 Hz. Raw force-time data were automatically processed by specialized software (T-Force System, Ergotech) with a 4^th^-order low-pass Butterworth filter showing no phase shift, and utilizing a 200 Hz cutoff frequency. The warm-up consisted of two attempts at 70% and 90% effort, with a 30-second rest between them. Maximal isometric force (MIF) and maximum rate of force development (RFDmax) were recorded, with RFDmax defined as the maximum slope in the force-time curve within 20 ms time intervals. Additionally, the average tangential slope of the force-time curve was measured over different time intervals (50, 100, 150, 200, and 400 ms from the onset of force production: RFD0–50, RFD0–100, RFD0–150, RFD0–200, and RFD0–400, respectively). The average value of each variable from the two attempts was recorded for further analysis.

#### Electromyography signal acquisition

After preparing the skin, a surface electromyography (EMG) electrode was placed on the VLA muscle of the right leg following SENIAM guidelines [24]. To maintain consistency in electrode placement across testing days, positions were recorded on transparent acetate sheets using anatomical landmarks and skin moles as references. EMG signals were recorded continuously during MVIC, progressive loading testing, and fatigue test using a parallel-bar, bipolar surface electromyographic sensor Trigno™ wireless EMG system, with an interelectrode distance of 10 mm, common mode rejection ratio > 80 dB, and bandwidth filter between 20 and 450 Hz ± 10% (Delsys Inc, Natick, MA). The baseline noise was < 5 μV peak-topeak and the sampling rate was 2,000 Hz. The raw data from the EMG were stored in digital format using EMG Works Acquisition software (Delsys Inc). From each isometric and dynamic contraction, the highest (over sliding windows of 500 ms with an overlap of 499 ms) root mean square (RMS) value was recorded. RMS values were normalized using the pre-training MVIC signal.

#### Progressive loading test

A progressive loading test on a Smith machine (Multipower Fitness Line, Peroga, Murcia, Spain) with no counterweight mechanism was performed to determine the individual load-velocity (L-V) relationship and the 1RM load in the SQ exercise. Subjects started from the upright position with the knees and hips fully extended, parallel feet and stance approximately shoulder-width apart, and the barbell resting across the back at the level of the acromion. Each subject descended in a continuous and controlled motion (~0.50–0.65 m · s^−1^) as low as possible (~35–40º knee flexion), then immediately reversed motion and raised back to the upright position. Unlike the eccentric phase, the concentric phase was always executed at maximal velocity. All data were collected with a linear velocity transducer (T-Force System Ergotech, Murcia, Spain), whose reliability has been reported elsewhere [25]. Participants warmed up with 6 repetitions of 20 kg, after which the load progressively increased in increments of 10 kg, then 5 kg, and finally, 2.5 kg when MPV dropped below 0.60 m · s^−1^ until MPV was less than 0.50 m · s^−1^. Three repetitions were performed for light loads (≥ 1.00 m · s^−1^), two for medium loads (1.00–0.80 m · s^−1^), and one for heavy loads (≤ 0.80 m · s^−1^). Interset recoveries ranged from 3 minutes (light loads) to 5 minutes (heavy loads). The repetition with the highest MPV for each load was used for analysis. MPV corresponds to the portion of the concentric action during which the measured acceleration is greater than the acceleration due to gravity (-9.81 m · s^−2^) [26]. The 1RM strength was calculated from the individual second-order load-velocity relationships calculating the absolute load that each subject could lift at 0.32 m · s^−1^ [27]. This load-velocity relationship was calculated with loads up to 90% 1RM (MPV < 0.50 m · s^−1^). In addition to the individual L–V relationship and the 1RM load, the following variables were examined: a) average MPV attained against all absolute loads common to pre-training and post-training (AV), b) average MPV attained against the absolute loads that were moved faster than 1 m · s^−1^ at pre-training (AV > 1), and c) average MPV attained against the absolute loads that were moved slower than 1 m · s^−1^ at pre-training (AV ≤ 1). These variables were analyzed to examine the potential adaptations achieved in different areas of the L–V relationship.

#### Fatigue test

Five minutes after the SQ progressive loading test, subjects were instructed to perform as many repetitions as possible with 70% of their 1RM until their MPV dropped below 0.50 m · s^−1^. The test was repeated after the completion of the study using the same absolute load as in the pre-training examination (i.e., 70% of baseline 1RM). The execution technique and equipment were similar to those described in the progressive loading test. The maximum number of repetitions (MNR) and the average MPV at the same number of repetitions before and after training (AV-MNR) were analyzed.

#### Determination of arterial occlusion pressure

Before the first session, the arterial occlusion pressure was determined. Subjects lay down for 10 minutes prior to testing. A 12 x 86 cm contoured cuff (VBM20-54-528, Medizintechnik, GmbH, Germany) featuring a smaller distal than proximal diameter to facilitate blood flow occlusion at lower pressures, was used. Subjects were seated with knees flexed at 90° (180° = full extension). A hand-held Doppler probe (Dopplex D900, Huntleigh Healthcare Ltd., UK) was placed on the tibialis posterior artery. The cuff was inflated to 70 mmHg using a manual inflator for each leg (VBM20-18-601-VBM20-18-602, respectively), then gradually increased by 10 mmHg increments until no pulse was heard in accordance with the recommendations of Sieljacks et al. [1]. Two measurements per leg were taken, with 5-minute rests between attempts, and the average value was recorded. If the difference between attempts exceeded 15 mmHg, a third measurement was taken.

### Resistance training program

The descriptive characteristics of the RT program are presented in Table 1. The four groups trained twice a week for 8 weeks, with 48–72 hours between sessions. Subjects performed SQ exercise with increasing relative intensity (55% to 65% 1RM), decreasing repetitions per set (10 to 6), 3 sets, and 2-minute rests between sets. Two groups followed MaxV protocols, where each repetition was performed at maximal velocity: one with blood flow restriction (MaxV-BFR) using a cuff on each leg placed proximally to the inguinal fold region of the exercised thigh, and the other protocol was conducted under the FF condition (MaxV-FF) condition. The other two groups were instructed to intentionally reduce their concentric velocity to half of the target MPV established for each training session, following the HalfV protocols. BFR pressure was set at 50% of arterial occlusion pressure as higher pressures showed no additional benefit when combined with heavier loads [3]. Subjects received real-time feedback on movement velocity in every repetition, measured using a linear transducer, ensuring velocities matched the prescribed %1RM. Relative loads were determined from the individual L–V relationships obtained from the progressive loading test in the SQ exercise for each subject (R^2^ = 0.99 ± 0.01). Therefore, for each training session, individual loads were adjusted to ensure that the corresponding MPV matched (within ± 0.03 m · s^−1^) the prescribed %1RM. A standardized warm-up preceded each training session: i) 5 minutes of jogging at a self-selected easy pace, ii) 2 x 10 repetitions of SQ (own body weight), iii) 4 x 6-6-4-1 repetitions of SQ (20 kg, 40%, 50%, and 55% of 1RM, respectively) for sessions 1–5; or iii) 4 x 6-6-4-1 repetitions of SQ (20 kg, 40%, 50%, and 60% of 1RM, respectively) for sessions 6–10; or iii) 4 x 6-6-4-2-1 repetitions of SQ (20 kg, 40%, 50%, 60% and 65% of 1RM, respectively) for sessions 11–16. Warm-up loads were lifted at maximal velocity for MaxV protocols and half-maximal velocity for HalfV protocols. However, all participants performed the heaviest warm-up load at the maximal velocity to determine the training session load. BFR pressure (50% arterial occlusion pressure) was applied 30 s before the first set and maintained during rest intervals, with subjects seated to keep the restriction constant, following standard BFR study protocols (5–10 minutes) [4, 6] within the typical restriction time commonly found in BFR studies (5–10 minutes) [28]. Subjects had to remain seated between each set to maintain the established pressure [1].

**TABLE 1:** Descriptive characteristics of the 8-week squat training program performed by the four experimental groups.

Scheduled	Session 1	Session 2	Session 3	Session 4	Session 5	Session 6	Session 7	Session 8
**Set × Rep**	3 x 10	3 x 10	3 x 10	3 x 10	3 x 10	3 x 8	3 x 8	3 x 8
**Target %1RM**	~55	~55	~55	~55	~55	~60	~60	~60

**Scheduled**	**Session 9**	**Session 10**	**Session 11**	**Session 12**	**Session 13**	**Session 14**	**Session 15**	**Session 16**

**Set × Rep**	3 x 8	3 x 8	3 x 6	3 x 6	3 x 6	3 x 6	3 x 6	3 x 6
**Target %1RM**	~60	~60	~65	~65	~65	~65	~65	~65


**Actually Performed**		**55% 1RM**		**60% 1RM**			**65% 1RM**	

**Average target MPV** (m∙s^−1^)		0.96 ± 0.06		0.90 ± 0.05			0.82 ± 0.05


**Actually Performed**	**Fastest-V** (m ∙ s^−1^)		**Slowest-V** (m ∙ s^−1^)		**Mean-V** (m ∙ s^−1^)		**Average Training Intensity (%1RM)**	

**HalfV-BFR**	0.53 ± 0.03		0.37 ± 0.02		0.45 ± 0.03		60.9 ± 1.3	
**MaxV-BFR**	0.86 ± 0.06		0.57 ± 0.08		0.73 ± 0.06		59.8 ± 1.4	
**HalfV-FF**	0.52 ± 0.04		0.38 ± 0.03		0.45 ± 0.03		60.9 ± 1.3	
**MaxV-FF**	0.87 ± 0.06		0.64 ± 0.07		0.76 ± 0.06		59.4 ± 3.7	
BFR × VEL p-value	0.70		0.13		0.35		0.79	
BFR p-value	0.94		0.02		0.33		0.74	
VEL p-value	< 0.001		< 0.001		< 0.001		0.07	

Data are mean ± standard deviation, n = 46. Only one exercise (full squat) was used in training. HalfV-BFR: protocol that trained at half-maximal concentric velocity with blood flow restriction (n = 10); MaxV-BFR: protocol that trained at maximal concentric velocity with blood flow restriction (n = 11); HalfV-FF: protocol that trained at half-maximal concentric velocity with free blood flow (n = 12); MaxV-FF: protocol that trained at maximal concentric velocity with free blood flow (n = 13). Average target MPV: this value represents the average intensity (%1RM) achieved during the training program; MPV: mean propulsive velocity; Fastest-V: Average of the fastest repetitions measured in each session; Slowest-V: Average of the slowest repetitions measured in each session; Mean-V: Average MPV attained during the entire training program; Mean Velocity Loss: Average velocity loss attained during the entire training program; Average training intensity: average intensity performed in each session; Average training intensity with a given %1RM: average intensity performed in each session with each of the loads used (55%, 60% or 65% 1RM).

### Statistical Analysis

Values are reported as mean ± standard deviation (SD). Test–retest reliability was assessed by the CV and intraclass correlation coefficient (95% CI) calculated using the one-way random effects model. Following the guidelines by Atkinson & Nevill [29], a CV < 10% was considered to represent acceptable absolute reliability. ICC values were classified according to Koo & Li [30] as poor (< 0.50), moderate (0.50–0.75), good (0.75–0.90), or excellent (> 0.90) reliability. At pre-training, all data were found to be normally distributed, as determined by the Shapiro-Wilk test. A 2 (FF vs. BFR) × 2 (MaxV vs. HalfV) repeated measures ANOVA was conducted to compare the different RT programs. Additionally, a 2 (FF vs. BFR) × 2 (MaxV vs. HalfV) × 2 (Pre vs. Post) repeated measures ANOVA was performed to analyze the differences in long-term effects between the four different resistance training programs. Bonferroni’s post hoc adjustments were applied when appropriate. Pre-post effect size (ES) values were calculated using Hedge’s g with pooled SD [31]. Changes were considered relevant when the difference score was at least ± 0.2 SD for each group. The ES was classified as small (0.2–0.5), moderate (0.5–0.8), or large (> 0.8) [32]. Statistical significance was established at the p ≤ 0.05 level. ES was calculated with Excel. The rest of the statistical analyses were performed using SPSS software version 22.0 (SPSS Inc., Chicago, USA).

## RESULTS

Training compliance was 100% for all sessions among the subjects who completed the intervention. The reliability values of the different tests conducted are shown in Table 2.

**TABLE 2:** Reliability values of different tests conducted in the present study.

Parameter	ICC (95% CI)	CV %
**US**		
ACSA_40_	0.989 (0.979–0.994)	2.7
ACSA_57.5_	0.991 (0.984–0.995)	2.1
ACSA_75_	0.980 (0.963–0.990)	3.0

**Dynamic**		
CMJ	0.996 (0.993–0.997)	1.8

**MVIC**		
MIF	0.950 (0.909–0.972)	7.0
RFD_max_	0.894 (0.788–0.947)	15.1
RFD0–50	0.841 (0.677–0.922)	23.9
RFD_0–100_	0.874 (0.744–0.938)	21.8
RFD_0–150_	0.905 (0.807–0.953)	16.9
RFD_0–200_	0.926 (0.849–0.964)	14.2
RFD_0–400_	0.915 (0.827–0.958)	12.4
RMS	0.965 (0.937–0.980)	13.7

ICC = intraclass correlation coefficient; CI = confidence interval; CV = coefficient of variation; ACSA_40_, ACSA_57.5_, and ACSA_75_ = anatomical cross-sectional area at 40%, 57.5%, and 75% of the femur length, respectively; CMJ: countermovement jump; MVIC: maximal voluntary isometric contraction; MIF: maximal isometric force; RFDmax: maximal rate of force development; RFD_0–50_: rate of force development from the onset of force production to 50 ms; RFD_0–100:_ from the onset of force production to 100 ms; RFD_0–150_: from the onset of force production to 150 ms; RFD_0–200_: from the onset of force production to 200 ms; RFD_0–400_: from the onset of force production to 400 ms; RMS: root mean square averaged from the vastus medialis and vastus lateralis muscles.

### Individual load-velocity relationship, 1RM, and vertical jump test

Adaptations induced by RT detected in dynamic SQ and vertical jump performance are displayed in Table 3. A significant “time effect” (*p* ≤ 0.001) was observed for all analyzed variables. No significant interactions were found between “BFR × time” or “VEL × time”. However, a significant “BFR × VEL × time” interaction (*p* = 0.05) was observed for CMJ height. HalfV-BFR and MaxV-FF experienced significant increases in CMJ height (*p* < 0.01). All protocols exhibited significant gains in 1RM strength (*p* < 0.001). Regarding changes in the L-V relationship, all protocols showed significant increases in AV, AV ≤ 1, and AV > 1 (*p* < 0.001). In addition, significant increases in MNR and AV-MNR were observed for all protocols (*p* < 0.001). MaxV-BFR resulted in significantly higher increases in MNR compared to MaxV-FF (*p* < 0.05).

**TABLE 3:** Changes in squat and jump performance from Pre-training to Post-training for each REP.

	HALFV-BFR			MAXV-BFR		

	Pre	Post	ES	Pre	Post	ES
1RM (kg)	104.8 ± 24.3	121.2 ± 26.0***	0.69	105.0 ± 17.4	125.3 ± 20.6***	0.86
AV (m· s^−1^)	0.96 ± 0.10	1.08 ± 0.08***	1.33	0.97 ± 0.08	1.12 ± 0.10***	1.67
AV ≤ 1 (m· s^−1^)	0.70 ± 0.05	0.84 ± 0.06***	2.18	0.70 ± 0.04	0.88 ± 0.10***	2.81
AV > 1 (m· s^−1^)	1.29 ± 0.10	1.39 ± 0.09***	1.09	1.28 ± 0.07	1.40 ± 0.07***	1.31
CMJ (cm)	36.6 ± 4.7	39.5 ± 4.0**	0.47	37.3 ± 6.4	38.4 ± 6.8	0.18
MNR	10.1 ± 2.8	19.6 ± 5.8***	1.87	9.1 ± 3.8	21.0 ± 7.4*** ^MF^	2.36
AV-MNR (m· s^−1^)	0.60 ± 0.03	0.76 ± 0.09***	2.05	0.59 ± 0.04	0.80 ± 0.15***	2.70


	**HALFV-FF**			**MAXV-FF**		

	**Pre**	**Post**	**ES**	**Pre**	**Post**	**ES**

1RM (kg)	109.0 ± 21.3	124.1 ± 22.7***	0.64	108.4 ± 27.0	122.4 ± 26.6***	0.59
AV (m· s^−1^)	0.98 ± 0.05	1.12 ± 0.08***	1.57	0.95 ± 0.09	1.07 ± 0.11***	1.35
AV ≤ 1 (m· s^−1^)	0.72 ± 0.03	0.88 ± 0.06***	2.51	0.70 ± 0.04	0.83 ± 0.08***	2.05
AV > 1 (m· s^−1^)	1.29 ± 0.06	1.40 ± 0.11***	1.21	1.26 ± 0.07	1.36 ± 0.13***	1.10
CMJ (cm)	36.8 ± 4.9	38.1 ± 4.6	0.21	37.1 ± 7.2	40.1 ± 7.8**	0.50
MNR	10.3 ± 4.1	18.4 ± 4.0***	1.61	8.4 ± 2.1	16.0 ± 6.1***	1.51
AV-MNR (m· s^−1^)	0.62 ± 0.04	0.76 ± 0.06***	1.81	0.61 ± 0.03	0.77 ± 0.09***	2.07


	***P*-value time effect**	***P*-value BFR × VEL × time**		***P*-value BFR × time**	***P*-value VEL × time**	

1RM (kg)	< 0.001	0.38		0.19	0.64	
AV (m· s^−1^)	< 0.001	0.29		0.58	0.97	
AV ≤ 1 (m· s^−1^)	< 0.001	0.11		0.31	0.97	
AV > 1 (m· s^−1^)	< 0.001	0.43		0.91	0.87	
CMJ (cm)	< 0.001	0.05		0.87	0.97	
MNR	< 0.001	0.47		0.17	0.63	
AV-MNR (m· s^−1^)	< 0.001	0.63		0.27	0.23	

Data are mean ± SD, n = 46. HALFV-BFR: half of the maximum voluntary velocity with blood flow restriction (n = 10); MAXV-BFR: maximum voluntary velocity with blood flow restriction (n = 11); HALFV-FF: half of the maximum voluntary velocity with free blood flow (n = 12); MAXV-FF: maximum voluntary velocity with free blood flow (n = 13). Sample size differed for MNR (HALFV-BFR: n = 9; MAXV-BFR: n = 11; HALFV-FF: n = 12; MAXVFF: n = 12) and AV-MNR (HALFV-BFR: n = 10; MAXV-BFR: n = 11; HALFV-FF: n = 12; MAXVFF: n = 12). 1RM: one-repetition maximal in full squat exercise; AV: average MPV attained against all absolute loads common to Pre- and Post-training; AV < 1: average MPV attained against absolute loads that were moved slower than 1 m · s^−1^ at Pre-training; AV > 1: average MPV attained against absolute loads that were moved faster than 1m · s^−1^ at Pre-training; CMJ: countermovement jump. MNR; maximal number of repetitions in the fatigue test; AV-MNR; average MPV in the fatigue test. ES: within-group effect size from pre- to post-training. Intragroup significant differences from Pre- to Post-training:

* *p* < 0.05,

** *p* < 0.01,

*** *p* <s 0.001. Significant differences with respect to MaxV-FF: ^MF^ *p* < .05.

### Maximal Voluntary Isometric Contraction

**FIG. 1:**
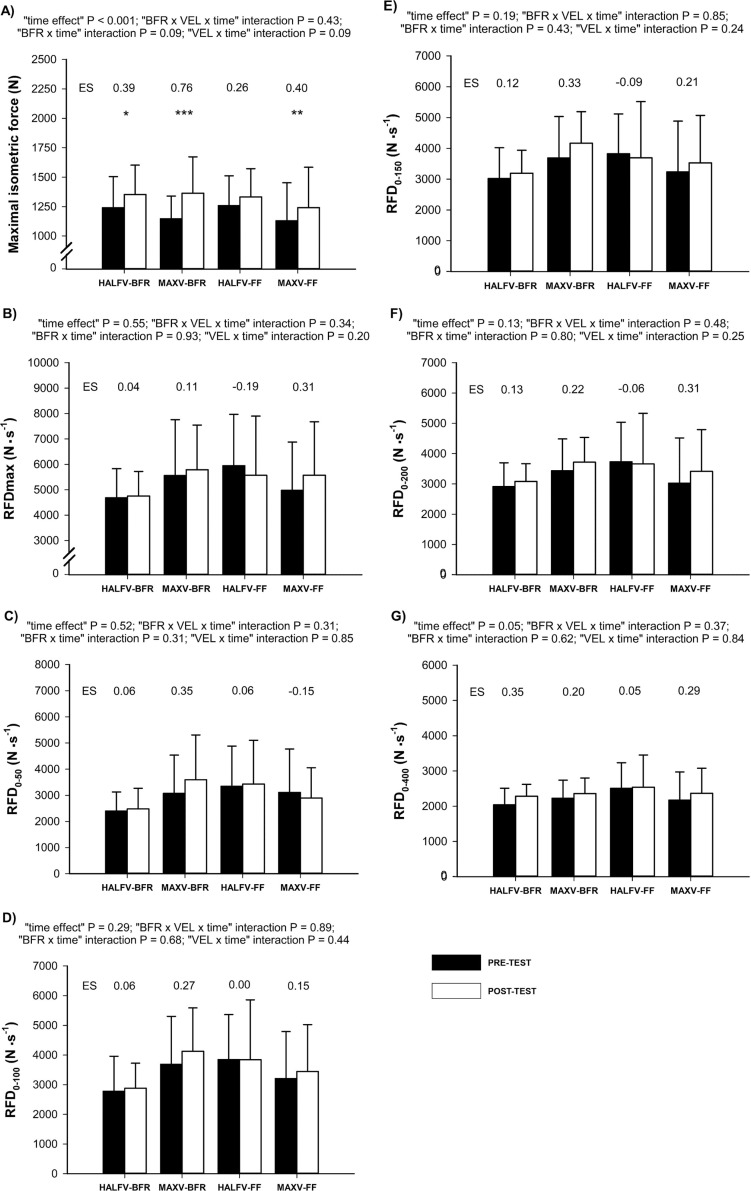
Changes produced from Pre- to Post-training for each group in MIF (A), RFDmax (B), rate of force development (C) from the onset of force production to 50ms (RFD_0–50_), (D) from the onset of force production to 100ms (RFD_0–100_), (E) from the onset of force production to 150ms (RFD_0–150_), (F) from the onset of force production to 200ms (RFD_0–200_), (G) from the onset of force production to 400ms (RFD_0–400_). ES: within-group effect size from Pre- to Post-training. Data are mean ± SD, n = 46. HalfV-BFR: half of the maximum voluntary velocity with blood flow restriction (n = 10); MaxV-BFR: maximum voluntary velocity with blood flow restriction (n = 11); HalfV-FF: half of the maximum voluntary velocity with free blood flow (n = 12); MaxV-FF: maximum voluntary velocity with free blood flow (n = 13). Sample size differed for RFDmax (HalfV-BFR: n = 9; MaxV-BFR: n = 11; HalfV-FF: n = 11; MaxV-FF F: n = 12), and RFD_0–50,_ RFD_0–100,_ RFD_0–150,_ RFD_0–200_, RFD_0–400_ (HalfV-BFR: n = 9; MaxV-BFR: n = 10; HalfV-FF: n = 11; MaxV-FF F: n = 12). Intragroup significant differences from Pre- to Post-training: *p < 0.05, **p < 0.01, *** *p* < 0.001.

Figure 1 displays the main results regarding MVIC. Significant time effects were only observed for MIF (*p* < 0.001) and RFD_0–400_ (*p* = 0.05). No significant interactions were found for any variable between “BFR × VEL × time”, “BFR × time”, and “VEL × time”. Significant increases were observed for BFR protocols (*p* < 0.001–0.05) and MaxV-FF (*p* < 0.01) in MIF.

### Muscle hypertrophy

**FIG. 2:**
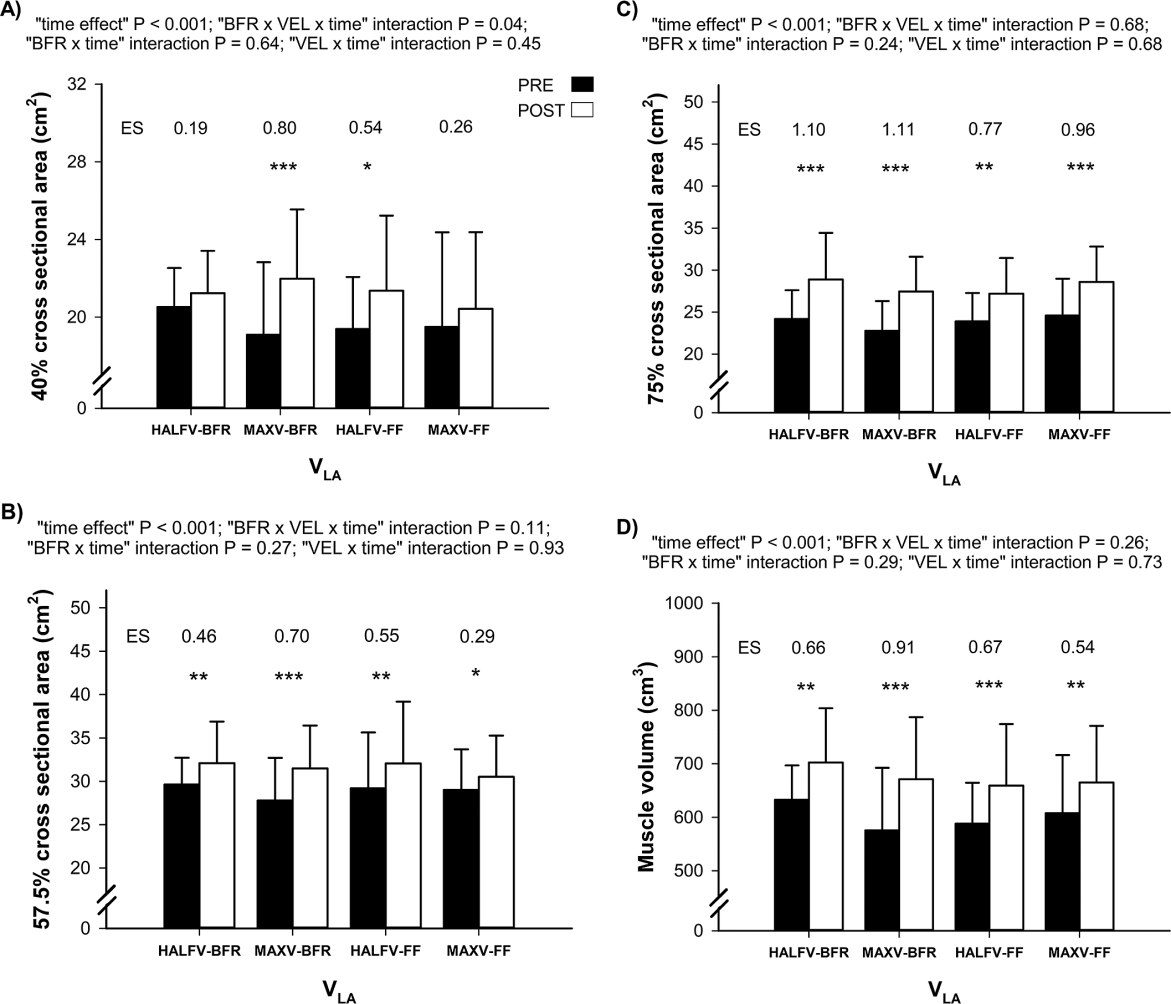
Changes in ACSA40 (A), ACSA57.5 (B), ACSA75 (C), and muscle volume (D) of VLA obtained from ultrasound images from Pre-training to Post-training for each group REP. Data are mean ± SD, n = 42. HalfV-BFR: half of the maximum voluntary velocity with blood flow restriction (n = 9); MaxV-BFR: maximum voluntary velocity with blood flow restriction (n = 11); HalfV-FF: half of the maximum voluntary velocity with free blood flow (n = 10); MaxV-FF: maximum voluntary velocity with free blood flow (n = 12). Sample size differed for ACSA75 and muscle volume (HalfV-BFR: n = 9; MaxV-BFR: n = 11; HalfV-FF: n = 9; MaxV-FF F: n = 12). Intragroup significant differences from Pre- to Post-training: * *p* < 0.05, ** *p* < 0.01, *** *p* < 0.001.

Adaptations in ACSA_40_, ACSA_57.5_, ACSA_75,_ as well as muscle volume, are shown in Figure 2. A significant “time” effect (*p* < 0.001) was observed for all analyzed variables. No significant interactions were noted for “BFR × time” and “VEL × time”. However, a “BFR × VEL × time” interaction was found for ACSA_40_ (*P* = 0.04). Significant improvements in ACSA_40_ were observed only for MaxVBFR (*p* < 0.001) and HalfV-FF (*p* < 0.05). All protocols exhibited significant increases in ACSA_57.5_ and ACSA_75_ (*P* = 0.001–0.05). Moreover, all protocols showed significant increases in muscle volume (*p* < 0.001–0.01), with the highest ES found for MaxV-BFR (ES = 0.91).

### Neuromuscular parameters

Table 4 presents the main findings regarding RMS in the progressive loading test, fatigue test, and MVIC test. No significant “time” effect or interactions of “BFR × VEL × time”, “BFR × time”, and “VEL × time” were observed for any of the variables analyzed, except a “VEL × time” interaction for AV > 1-RMS. HalfV-BFR demonstrated significant increases in AV-RMS (*p* < 0.05), AV ≤ 1-RMS (*p* < 0.05), AV > 1-RMS (*p* < 0.05), and MVIC-RMS (*p* < 0.05). In addition, significantly higher increases were observed in HalfV-BFR compared to HalfV-FF for MVIC-RMS (*p* < 0.05).

**TABLE 4:** Changes in neuromuscular parameters from Pre-training to Post-training for each REP

	HALFV-BFR			MAXV-BFR		

	Pre	Post	ES	Pre	Post	ES
AV-RMS (%)	102.7 ± 21.3	123.0 ± 29.2*	0.64	127.2 ± 25.6	124.6 ± 32.5	−0.08
AV ≤ 1-RMS (%)	104.1 ± 20.0	125.2 ± 30.1*	0.59	128.3 ± 24.5	125.4 ± 32.3	−0.08
AV > 1-RMS (%)	102.5 ± 25.1 ^MB^	123.4 ± 33.0*	0.67	129.8 ± 30.2	120.4 ± 32.8	−0.30
MNR-RMS (%)	106.7 ± 22.6	125.5 ± 26.8	0.49	130.2 ± 19.3	129.3 ± 27.5	−0.02
MVIC-RMS (%)	100.0 ± 0.0	118.8 ± 38.5*^HF^	0.87	100.0 ± 0.0	105.4 ± 22.0	0.25

	**HALFV-FF**			**MAXV-FF**		

	**Pre**	**Post**	**ES**	**Pre**	**Post**	**ES**

AV-RMS (%)	109.6 ± 34.3	108.0 ± 34.0	−0.05	117.7 ± 31.1	110.9 ± 27.0	−0.22

AV ≤ 1-RMS (%)	112.6 ± 38.5	112.1 ± 37.9	−0.01	126.5 ± 34.8	129.2 ± 45.1	0.08
AV > 1-RMS (%)	104.7 ± 29.0	102.0 ± 28.6	−0.09	114.0 ± 27.9	103.6 ± 24.6	−0.34
MNR-RMS (%)	108.9 ± 36.8	106.3 ± 43.5	−0.07	127.2 ± 35.2	128.2 ± 50.3	0.03
MVIC-RMS (%)	100.0 ± 0.0	87.6 ± 19.2	−0.58	100.0 ± 0.0	104.7 ± 29.7	0.22


	***P*-value time effect**	***P*-value BFR × VEL × time**		***P*-value BFR × time**	***P*-value VEL × time**	


AV-RMS (%)	0.59	0.30		0.13	0.10	
AV ≤ 1-RMS (%)	0.30	0.17		0.43	0.28	
AV > 1-RMS (%)	0.92	0.20		0.16	0.03	
MNR-RMS (%)	0.56	0.40		0.48	0.56	
MVIC-RMS (%)	0.35	0.09		0.08	0.83	

Data are mean ± SD, n = 42. HALFV-BFR: half of the maximum voluntary velocity with blood flow restriction (n = 10); MAXV-BFR: maximum voluntary velocity with blood flow restriction (n = 9); HALFV-FF: half of the maximum voluntary velocity with free blood flow (n = 12); MAXV-FF: maximum voluntary velocity with free blood flow (n = 11). Sample size differed for AV-RMS and AV > 1-RMS (HALFV-BFR: n = 9; MAXV-BFR: n = 9; HALFV-FF: n = 12; MAXVFF: n = 10), for AV < 1-RMS (HALFV-BFR: n = 9; MAXV-BFR: n = 10; HALFV-FF: n = 12; MAXVFF: n = 11) and MNR-RMS (HALFV-BFR: n = 6; MAXV-BFR: n = 7; HALFV-FF: n = 8; MAXVFF: n = 9). AV-RMS: root mean square averaged from the vastus medialis and vastus lateralis muscles in the MPV attained against all absolute loads common to Pre- and Post-training; AV < 1-RMS: root mean square averaged from the vastus medialis and vastus lateralis muscles in the MPV attained against absolute loads that were moved slower than 1 m · s^−1^ at Pre-training; AV > 1- RMS: root mean square averaged from the vastus medialis and vastus lateralis muscles in the MPV attained against absolute loads that were moved faster than 1m · s^−1^ at Pre-training; MNR-RMS: root mean square averaged from the vastus medialis and vastus lateralis muscles in the fatigue test; MVIC-RMS: root mean square averaged from the vastus medialis and vastus lateralis muscles in the maximal voluntary isometric contraction test. ES: within-group effect size from pre- to post-training. Intragroup significant differences from Pre- to Post-training:

* *p* < .05. Significant differences with respect to MaxV-BFR:

MB *p* < 0.05. Significant differences with respect to HalfV-FF:

HF *p* < 0.05.

### Training program

**FIG. 3:**
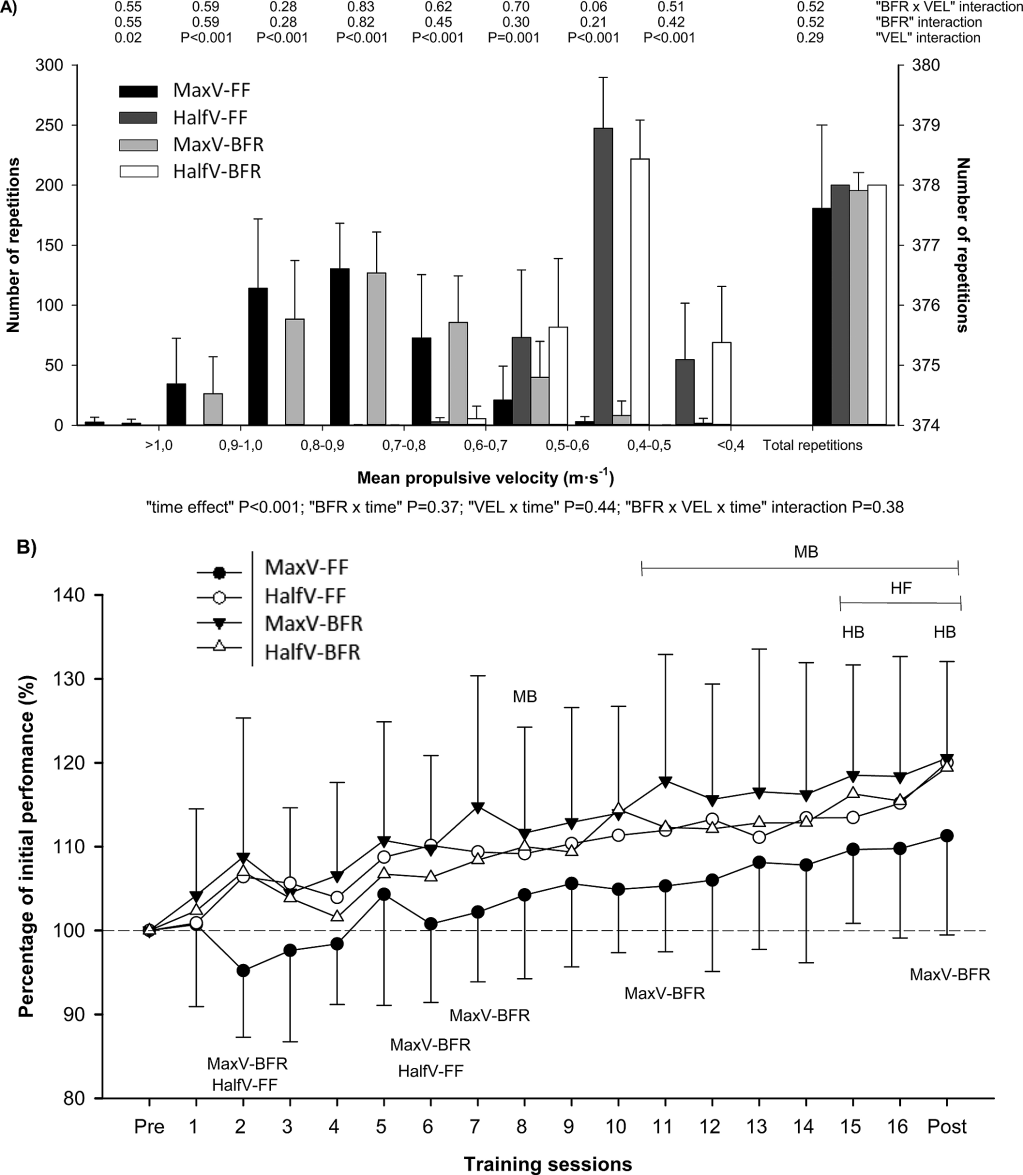
(A) Number of repetitions performed in each velocity range and total number of repetitions completed by each training group. (B) Evolution of the performance in the full-squat exercise in each training session expressed as a percentage of the initial pretraining level for each experimental group. HB (HalfV-BFR), MB (MaxV-BFR), and HF (HalfV-FF) indicate the session from which the respective group attained significant improvements (*p* < 0.05) compared with their pretraining values. Significant differences (*p* < 0.05) with respect to MaxV-BFR: MaxV-BFR; Significant differences (*p* < 0.05) with respect to HalfV-FF: HalfV-FF. Data are mean ± SD, n = 46. HalfV-BFR: half of the maximum voluntary velocity with blood flow restriction (n = 10); MaxV-BFR: maximum voluntary velocity with blood flow restriction (n = 12); HalfV-FF: half of the maximum voluntary velocity with free blood flow (n = 12); MaxV-FF: maximum voluntary velocity with free blood flow (n = 13).

The main characteristics of the RT programs are detailed in Table 1. No significant “BFR × VEL” interactions were observed for any variable. However, a “BFR” effect was noted for Slowest-V, where FF protocols displayed higher Slowest-V values than BFR protocols (*P* = 0.02). Additionally, “VEL” effects were identified for Fastest-V, Slowest-V, and Mean-V. MaxV protocols showed higher Fastest-V, Slowest-V, and Mean-V compared to HalfV protocols. The repetitions performed in different velocity ranges and total repetitions are depicted in Figure 3A. No significant “BFR × VEL” and “BFR” interactions were observed for any velocity. Nevertheless, a “VEL” effect was evident across all velocity ranges except in total repetitions. A greater number of repetitions were executed with velocities ranging from 0.6 to > 1.0 m·s^−1^ for MaxV protocols (p < 0.001–0.02). Conversely, a greater number of repetitions were performed with velocities ranging from < 0.4 to 0.6 m·s^−1^ for HalfV protocols (p < 0.001–0.001). Figure 3B illustrates the evolution of 1RM (expressed as a percentage of pre-training values) in each training session for all REPs, based on individual L-V relationships. A significant “time” effect was found, while no “BFR × VEL × time”, “BFR × time” and “VEL × time” interactions were detected.

## DISCUSSION

The main findings of this study were: i) all training groups showed similar improvements in SQ performance; ii) in the MVIC test, all groups demonstrated improvements in MIF, except for the HalfV-FF group; iii) only the HalfV-BFR and MaxV-FF groups exhibited enhancements in CMJ height; iv) HalfV-BFR resulted in positive neuromuscular adaptations, as demonstrated by the increased RMS; and v) MaxV-BFR and Half-FF induced greater distal hypertrophy (ACSA_40_), with MaxV-BFR showing the highest ES in muscle volume.

MaxV protocols completed more repetitions at higher velocities (> 0.6 m · s^−1^), resulting in faster velocities during training sessions compared to HalfV protocols. The fatigue experienced by MaxV protocols during training was moderate, as indicated by the velocity loss (VL) values (MaxV-BFR: 23.8% and MaxV-FF: 17.6%). These VL magnitudes correspond to approximately half-maximal repetitions [25]. The use of BFR may have influenced the descriptive characteristics of the training, leading to lower velocities and higher VL during training sessions as has been observed in the literature with the same intensity and volume [17]. BFR-RE causes greater peripheral fatigue and reduces the time to failure [33–36], due to accelerated accumulation of inorganic phosphate [37, 38]. Regarding fatigue in the HalfV protocols, VL is not a valid tool to assess fatigue during training when each repetition is not performed at maximal velocity. This is explained by the fact that VL measures the difference between the fastest and the last repetition due to fatigue. However, HalfV protocols aim to maintain the same velocity (half-maximal) across all repetitions. Therefore, comparing both velocity conditions is not feasible.

After the 8-week (16 sessions) training period, all groups demonstrated similar improvements across all SQ performance variables. In addition to assessing the change in 1RM strength, we evaluated the changes in velocity developed against all loads (AV), ‘light’ loads (AV > 1), and ‘heavy’ loads (AV ≤ 1) common to the pre- and posttraining sessions, aiming to analyze the extent to which the four RT programs affected different parts of the L-V relationship. Pareja-Blanco et al. [15] similarly did not observe a “group × time” interaction in 1RM and AV between MaxV and HalfV under FF conditions. Nevertheless, they noted a tendency for greater strength gains in the SQ exercise for the MaxV protocol. Consistent with this observation, in our study, the HalfV-FF protocol was the only protocol that did not yield significant improvements in MIF. Slow lifting with light-to-moderate loads and a moderate level of effort under FF conditions may indeed fail to maximize strength gains due to several factors, including the insufficient recruitment of high-threshold motor units. Faster movements against a given load activate more muscle fibers, particularly the high-threshold motor units responsible for generating maximal force [39]. This recruitment pattern is crucial for stimulating the neural adaptations necessary for significant strength gains [40].

In our study, the use of the BFR methodology did not significantly affect the gains observed in SQ performance compared to FF protocols. It is noteworthy that a recent study using maximal concentric velocity at moderate intensities (55–70% 1RM) reported that BFR did not yield greater strength gains than FF-RT, consistent with our findings [16]. However, another recent study suggested that combining BFR with maximal concentric velocity can produce greater strength gains than the MaxV-FF condition [17]. The discrepancies between these two studies may be attributed to methodological differences, primarily in how training volume was standardized. In the Sánchez-Valdepeñas et al. [16] study, volume was matched based on VL, requiring participants to adjust repetitions to achieve the same percentage of VL; consequently, subjects in the BFR protocols performed fewer repetitions than those in the FF protocols. In contrast, Cornejo-Daza et al. [17] matched the volume by the number of repetitions, resulting in a significantly greater VL in the BFR protocols compared to FF conditions. In the present study, although the differences were not statistically significant, the MaxV-BFR protocol exhibited the greatest ES in strength gains. However, this was not observed in the HalfV-BFR protocol. Therefore, we cannot conclude that BFR is the mechanism responsible for these improvements. By contrast, in studies in which BFR-RT was performed under controlled conditions, some investigations have reported superior gains compared with low-load FF-RT [7, 8]. Nevertheless, a broader review of the literature indicates that high-load RT is generally more effective than low-load BFR-RT in promoting muscle strength gains [9], suggesting that BFR-RT may not always represent the optimal approach for maximizing strength. Despite these considerations, we found that MaxV-BFR showed greater improvements in MNR than MaxV-FF under moderate load conditions. Similar results regarding muscular endurance with BFR conditions and moderate loads (approximately 50% 1RM) have been observed in previous research [7]. These findings emphasize the need for additional investigation in this area.

A “BFR × VEL × time” interaction (*P* = 0.05) was found for CMJ, where HalfV-BFR and MaxV-FF achieved significant improvements in CMJ height following the RT program. Similarly, Pareja-Blanco et al. [15] observed a “group × time” interaction in CMJ, with MaxV demonstrating greater improvements than HalfV under FF conditions. These results align with those for RFD, in which the HalfV-FF protocol exhibited the lowest ES values for RFD at different time intervals (Figure 1). There are several potential explanations for why the MaxV-FF protocol improved CMJ height while the HalfV-FF protocol did not. Firstly, each repetition in the MaxV protocol may lead to greater recruitment of fast-twitch muscle fibers [41, 42]. Additionally, changes in MHC isoform composition, which influence muscle contractile properties, could contribute to the observed differences [43]. Furthermore, increases in tendon-aponeurosis stiffness, enhancing force transmission during explosive movements, may also play a role [44]. Finally, an increased calcium sensitivity of the contractile apparatus [45] could further enhance performance in the MaxV-FF protocol. Likewise, it is worth noting that strength gains are specific to the training stimulus. Higher training velocities better mimic the demands of explosive movements such as the CMJ. Moreover, lifting slowly reduces the utilization of elastic energy and the stretch-shortening cycle, which are important mechanisms for maximizing rapid force production [46, 47].

Despite the potential advantages of high-velocity lifting, our findings indicate that MaxV with BFR cuffs may counter the positive effect of maximal-velocity lifting on CMJ performance, consistent with previous research using similar methodology (intensity, number of repetitions, rest) [17]. However, unlike the FF protocols, HalfV-BFR showed a significant improvement in CMJ height. This could be explained by the positive neuromuscular adaptations observed for HalfV-BFR, such as increased muscle excitability at different loading conditions (i.e. increased AV ≤ 1-RMS, AV > 1-RMS, AV-RMS, and MVIC-RMS). Increased muscle excitability following long-term training programs may be due to increased motor units recruitment and/or firing frequency [48]. Several previous studies also demonstrated higher muscle excitation with BFR compared to FF exercises when matching the number of repetitions per set without reaching muscle failure and intentionally slow movement velocities [49–51]. These findings emphasize the intricate interplay between BFR, lifting velocity, and neuromuscular adaptations, underscoring the necessity for further research to optimize the integration of these factors in RT protocols aimed at optimizing performance outcomes.

An important finding from this study was that, unlike the CMJ, MaxV-BFR and HalfV-FF protocols induced greater muscle hypertrophy compared to others, as evidenced by the “BFR × VEL × time” interaction in ACSA_40_ (P = 0.04), with MaxV-BFR resulting in the highest muscle volume gains (ES = 0.91). The hypoxic muscular environment generated during BFR may induce high metabolic stress, thereby activating mechanisms for muscle growth [52]. In addition, it has been shown that MaxV-BFR maximize muscle hypertrophic gains compared to MaxV-FF [16, 17]. One potential mechanism for this enhanced hypertrophy with BFR is the recruitment of fast-twitch fibers [53, 54], which are more susceptible to exercise-induced crosssectional area gains than slow-twitch fibers [53], since ischemia can inhibit the twitch responses of type I muscle fibers [55]. Moreover, lifting loads at higher velocities may potentiate this mechanism, though this hypothesis requires further research. Regarding the FF condition, a systematic review with meta-analysis indicated that hypertrophic outcomes are similar across repetition durations ranging from 0.5 to 8 s up to concentric muscular failure [56]. The results of this study indicated that HalfV-FF resulted in greater muscle hypertrophy than MaxV-FF, especially in the most distal ACSA. This may be due to the longer TUT accumulated by HalfV-FF in each repetition. Longer TUT may play a similar role to that played by BFR condition. Evidence suggests that intentionally slowing down the movement tempo of a single repetition can increase muscle activation for a given number of repetitions [57, 58]. Hypothetically, increased muscle activity combined with longer TUT could positively mediate intracellular anabolic signalling, promoting a greater hypertrophic response [56]. In this regard, Tanimoto & Ishii [59] indicated that slowing down the tempo, while maintaining the same intensity and volume, resulted in significantly greater hypertrophy than a faster tempo. Taken together, our findings suggest that MaxVBFR and HalfV-FF protocols may be particularly effective for stimulating muscle growth.

When interpreting our findings, it is important to consider various limitations. Firstly, the participants’ energy and protein intake were not monitored in the study, which could have affected the RT adaptations. Secondly, caution is needed when interpreting the results regarding the RFD due to the limited reliability of our RFD measurements, as indicated by CVs ranging from 12.4% to 23.9%. Additionally, given the small number of subjects, we cannot rule out a type II error when comparing the four training types. Lastly, the same number of repetitions with the same intensity could exhibit variability in the level of effort experienced between subjects with different strength levels and training backgrounds. Furthermore, the level of discomfort experienced during the BFR training sessions was not assessed, although the cuff width used was specifically selected to be appropriate for the execution of the SQ exercise. Finally, since it has been shown that men and women respond differently to RT stimuli [60, 61, 62], mixing both sexes may be a confounding variable. For this reason, only resistance-trained men were included in this study. Future studies should replicate this study in resistance-trained women.

## CONCLUSIONS

The main findings of this study were that, despite the differences between protocols in mechanical performance during the training sessions, all training groups exhibited similar improvements in SQ performance. However, improvements in maximal isometric strength were observed in all groups except for the HalfV-FF. Interestingly, only the HalfV-BFR and MaxV-FF protocols led to enhancements in CMJ performance. Additionally, the HalfV-BFR was the sole protocol that resulted in positive neuromuscular adaptations. Moreover, the MaxV-BFR and HalfV-FF protocols were the only ones that induced hypertrophy in the distal ACSA. Therefore, from an applied perspective, practitioners should consider that there seems to be no clear advantage of using BFR in healthy populations over FF if the goal is to improve performance.
